# Supplementary material for: On Structural and Molecular Order in Cellulose Acetate Butyrate Films
Source: Polymers (Basel). 2023 May 6;15(9):2205. doi: 10.3390/polym15092205 (PMC10181278; doi:10.3390/polym15092205)
Supplement: Supplementary file 1 [file polymers-15-02205-s001.zip › polymers-2172164-supplementary.pdf]

Supplemental to: On structural and molecular order in cellulose acetate butyrate films

A schematic presentation of the film making procedure is found in Figure S1.

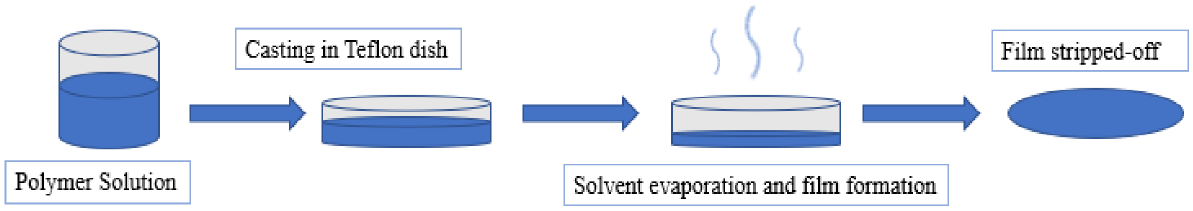

Figure S1. The solvent casting method for polymer film production.

FT-IR spectra of CAB-171-15 and CAB-551-0.01, the lowest and the highest butyrate substituted samples respectively, are found in Figure S2.

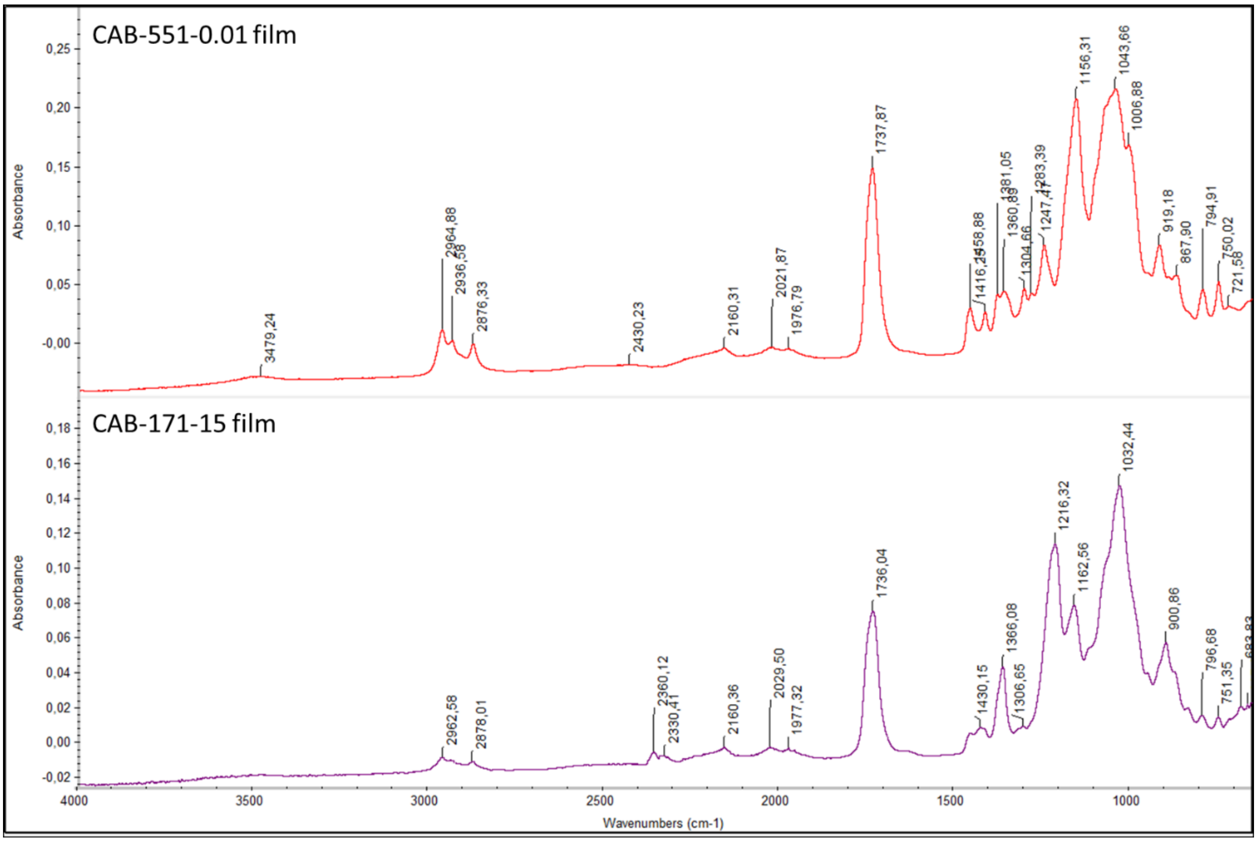

Figure S2. FT-IR spectra of CAB-171-15 and CAB-551-0.01, the lowest and the highest butyrate substituted samples respectively.
